# Generalizability of High Frequency Oscillation Evaluations in the Ripple Band

**DOI:** 10.3389/fneur.2018.00510

**Published:** 2018-06-28

**Authors:** Aaron M. Spring, Daniel J. Pittman, Yahya Aghakhani, Jeffrey Jirsch, Neelan Pillay, Luis E. Bello-Espinosa, Colin Josephson, Paolo Federico

**Affiliations:** ^1^Department of Clinical Neurosciences, University of Calgary, Calgary, AB, Canada; ^2^Hotchkiss Brain Institute, University of Calgary, Calgary, AB, Canada; ^3^Seaman Family MR Research Centre, Foothills Medical Centre, Calgary, AB, Canada; ^4^Department of Medicine, University of Alberta, Edmonton, AB, Canada; ^5^Department of Paediatrics, University of Calgary, Calgary, AB, Canada; ^6^Department of Radiology, University of Calgary, Calgary, AB, Canada

**Keywords:** high frequency oscillations (HFOs), generalizability, generalizability theory, interrater reliability, interrater variability, epilepsy, intracranial electroencephalography (iEEG)

## Abstract

**Objective:** We examined the interrater reliability and generalizability of high-frequency oscillation (HFO) visual evaluations in the ripple (80–250 Hz) band, and established a framework for the transition of HFO analysis to routine clinical care. We were interested in the interrater reliability or epoch generalizability to describe how similar the evaluations were between reviewers, and in the reviewer generalizability to represent the consistency of the internal threshold each individual reviewer.

**Methods:** We studied 41 adult epilepsy patients (mean age: 35.6 years) who underwent intracranial electroencephalography. A morphology detector was designed and used to detect candidate HFO events, lower-threshold events, and distractor events. These events were subsequently presented to six expert reviewers, who visually evaluated events for the presence of HFOs. Generalizability theory was used to characterize the epoch generalizability (interrater reliability) and reviewer generalizability (internal threshold consistency) of visual evaluations, as well as to project the numbers of epochs, reviewers, and datasets required to achieve strong generalizability (threshold of 0.8).

**Results:** The reviewer generalizability was almost perfect (0.983), indicating there were sufficient evaluations to determine the internal threshold of each reviewer. However, the interrater reliability for 6 reviewers (0.588) and pairwise interrater reliability (0.322) were both poor, indicating that the agreement of 6 reviewers is insufficient to reliably establish the presence or absence of individual HFOs. Strong interrater reliability (≥0.8) was projected as requiring a minimum of 17 reviewers, while strong reviewer generalizability could be achieved with <30 epoch evaluations per reviewer.

**Significance:** This study reaffirms the poor reliability of using small numbers of reviewers to identify HFOs, and projects the number of reviewers required to overcome this limitation. It also provides a set of tools which may be used for training reviewers, tracking changes to interrater reliability, and for constructing a benchmark set of epochs that can serve as a generalizable gold standard, against which other HFO detection algorithms may be compared. This study represents an important step toward the reconciliation of important but discordant findings from HFO studies undertaken with different sets of HFOs, and ultimately toward transitioning HFO analysis into a meaningful part of the clinical epilepsy workup.

## Introduction

In the treatment of drug-resistant focal epilepsy, the localization and removal of the region generating the seizures is critical to the successful elimination of seizures. It has been proposed that this region—the so-called epileptogenic zone (EZ)—may be identified using high frequency oscillations (HFOs) (1, 2), or interictal epileptiform discharges (IEDs) occurring simultaneously with HFOs (HFO+IEDs) (3, 4). Extensive research has been performed with regards to the characteristics of these HFOs and their correlation with epileptogenicity and the EZ. Nonetheless, the lack of generalizability and comparability between the various studies remains an obstacle to the implementation of HFOs prospectively in clinical practice (5).

There is clear and mounting evidence of a correlation between the EZ and HFOs. A meta-analysis revealed a significant correlation between the resection of tissue exhibiting HFOs and the absence of seizures following surgery (6). However, the findings of the individual retrospective studies within the meta-analysis, as well as subsequent studies, have varied substantially. Some found that the resection of HFOs in the ripple range (~80–250 Hz) correlated more strongly with positive outcomes than did the resection of HFOs in the fast ripple range (FR; ~250–500 Hz) (1), while others found that the opposite was true (7). There were studies that found a significant effect of resecting either ripples or FRs (2, 8), and more still that did not find any significant effect for either (9, 10). One study identified ripples to be superior at positive prediction, and FRs at negative prediction (11). More recently, it was shown that in some brain regions, the amplitudes or rates of HFOs are insufficient to distinguish the EZ from baseline activity (12).

There is also contradicting data regarding the role of HFOs occurring in isolation or those co-occurring with IEDs. There is some evidence that resection of HFO+IEDs are correlated with positive outcomes, while that of HFOs in isolation are not (13). There is also evidence that all HFOs are correlated with the EZ regardless of morphology (14), and other studies still have aimed to exclude HFOs generated by filtering IEDs and other sharp transients (15, 16). A recent study also provided evidence that the co-occurrence of ripples and FRs would be more useful than either alone in delineating the EZ (17), though this has yet to be reported in other studies.

Therefore, while there is overwhelming evidence confirming that HFOs are correlated with the EZ, it remains unclear which HFO characteristics would be most useful in prospectively identifying the EZ for surgical resection. Though prospective studies are ongoing (9, 18), a Cochrane review recently cited lack of evidence in concluding that “no reliable conclusions can be drawn regarding the efficacy of using HFOs in epilepsy surgery decision making at present” (19). Indeed, the different findings of all of these studies should first be reconciled.

That the results from the many studies undertaken in this field differ is not surprising – these studies have been undertaken using variable definitions of HFOs, methods of detection, and datasets. Many have relied upon visual review by one (3, 4, 14, 20–27) or two (1, 15, 16, 28–41) experts, and up to four experts have been used in rodents (42). However, while it has been shown that experts have moderate to strong agreement with regards to classifying candidate events as neural or artefactual in origin (43), it has also been shown that they have poor interrater reliability for classifying candidate events as HFOs (44) or gamma oscillations (45) in the first place.

In an effort to decrease the subjectivity and increase the speed of HFO identification, several automated or semi-automated detection algorithms have been developed. Some have been used in conjunction with expert review to obtain a set of HFO markings (43, 46–51), but many have been developed for use in isolation.

Several features have been proposed to quantify high frequency oscillatory activity, including amplitude threshold (52) or envelope (53), line length (33, 45), instantaneous frequency (33), conventional energy (45, 54), wavelet transforms (32), Stockwell entropy (11), non-harmonicity (50) and Teager energy (55). These features may be considered alone, or may be incorporated into a machine learning algorithm (33). Other algorithms have been developed based on pattern matching (23, 34, 42, 53); integration of data from multiple channels (11, 53); empirical mode decomposition (56); Gabor atoms (41); Gaussian mixture model clustering (57); topographic analysis (16, 51); independent component analysis (51); and integration of a rigorous artifact rejection step following HFO detection (43).

The performance of these algorithms may only be evaluated against some determined baseline. In theory, the ideal algorithm would be able to identify HFOs that perfectly delineate the EZ in prospective studies. In practice, this is not a widely feasible comparison, and even comparisons with retrospective surgical outcomes (11, 50) are rarely performed, so existing benchmarks are often used. Whether these benchmarks are other algorithms (43, 52), visual review (16, 32–34, 36, 41), or some combination thereof, the result is the same—the comparison of an algorithm against a set of markings that itself has not been validated in a generalizable, reliable manner, and therefore, a comparison of unknown clinical significance.

In the present study, we seek to establish a protocol for overcoming this critical limitation of both visual and algorithmic HFO identification. To accomplish this, we turn to a statistical framework called generalizability theory. The use of generalizability theory has been commonplace in the education literature for decades, but has only recently been implemented in neurology (58). It not only allows the characterization of interrater reliability in a manner that is less subject to bias than traditional metrics (59), but also predicts how changing the sample size could affect the generalizability (60). It may be used to determine how to create a dataset that would achieve strong generalizability in practice.

The present study makes use of generalizability theory to establish a framework for optimizing visual HFO markings, in order to reconcile the findings from diverse HFO studies and facilitate the transition of HFO evaluation to routine clinical care.

## Methods

This study was approved by our local Research Ethics Board. Forty-one consecutive adult patients (mean age: 35.6 years) were recruited, all with drug-resistant focal epilepsy and undergoing intracranial video-EEG monitoring (iVEM) at high sampling rates (1,000–2,000 Hz) for possible surgical candidacy at our epilepsy center. All patients were included in the study, regardless of the types or locations of the implanted intracranial electrodes, or of the type or presumed localization of the epileptogenic activity.

Data were initially processed as per the methodology detailed in Spring et al. (44). Twenty minutes of iEEG data were selected, filtered (80–250 Hz), derived (bipolar or Laplacian), and normalized (sliding 1 s root-mean-square). As in the previous study, the data were selected as close as possible to midnight, as close as possible to the fifth day postimplantation, in order to reduce artifact. No preference was given to stage of sleep or wakefulness due to the lack of concurrent scalp EEG recordings. Notably, this study focuses on HFOs in the ripple band (80–250 Hz), and the terms HFOs and ripples are used synonymously throughout this text, except where noted otherwise.

Three types of events were algorithmically detected from the normalized data: candidate HFO events, low-threshold HFO events, and distractor events. From each dataset (patient), 64 events of each type were pseudorandomly selected, resulting in a total of 7,872 events (64 events per type × 3 event types × 41 patients = 7,872 events). Filtered data (80–250 Hz) were presented to six visual reviewers as a series of epochs (250 ms), each containing the entirety of one event. Three seconds of unfiltered data were also provided simultaneously, and included the same 250 ms epoch, as well as the preceding and following 1,375 ms of data. In each case, the data presented included the target channel (in which the event was detected), the two nearest neighboring channels, and four channels randomly selected from those remaining.

**Figure 1:**
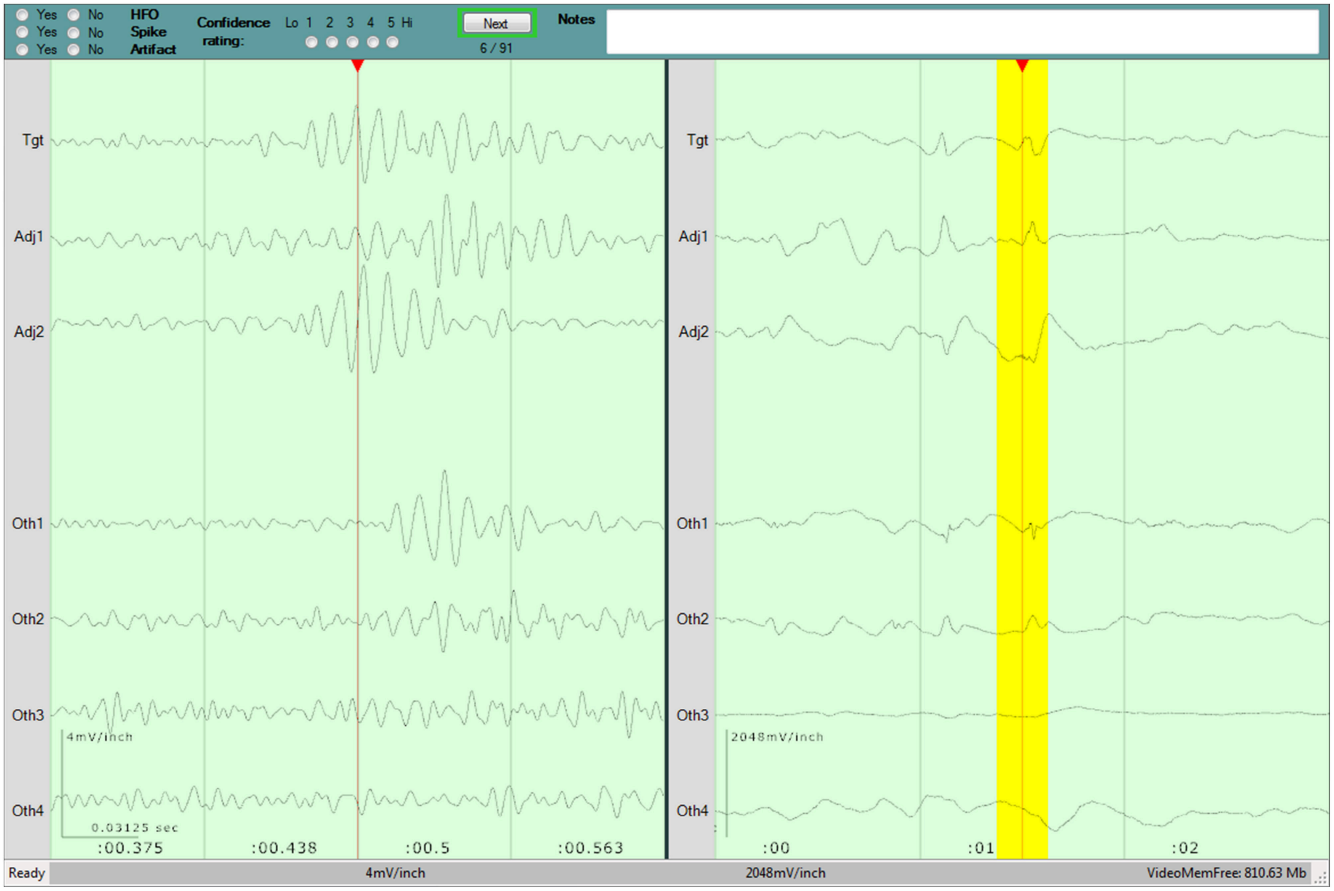
Screenshot of the program used for the visual review component of the study. Three seconds of raw data is shown in the right pane. Two hundred and fifty milliseconds of filtered data is shown in the left pane, and the corresponding raw data is highlighted in yellow. The top pane contains the evaluation form for the current Epoch, as well as the current progress. A detailed description of the evaluation program is available in our previous work (44).

A detailed overview of the evaluation program and process are published in our previous work (44), and a screenshot of the program is shown in Figure 1. The six reviewers (YA, JJ, NP, LB, CJ, PF) hailed from two epilepsy centers, and had varying degrees of experience evaluating HFOs, as detailed previously (44). They were instructed to identify HFOs that stood out from the surrounding baseline for at least 3 consecutive cycles. They also had the opportunity to mark the presence of any artifacts that they believed affected the presence or interpretation of an HFO.

### HFO rating

Each reviewer registered an HFO rating for each epoch. The rating was on a scale from −5 to +5, with the magnitude corresponding to the given confidence rating (1–5, with 5 indicating complete certainty), and the sign corresponding to the HFO evaluation of present (+) or absent (−). This rating reflects the likelihood of each epoch containing an HFO, as determined by each reviewer, and enables a more robust comparison of the relative “stringency” of the reviewers. In essence, stringency describes the probability of a reviewer positively marking an HFO of a given threshold. For example, a more stringent reviewer would typically assign lower ratings than a less stringent colleague, as they have a higher internal threshold for marking HFOs.

### Evaluation time

The evaluation program recorded the time each reviewer spent on each epoch evaluation. The mean evaluation time was then calculated for each reviewer, before and after eliminating outliers (lower and upper 5% of each reviewer's times).

### Generalizability studies

An initial generalizability study (G-study) was performed to characterize the observed interrater reliability and generalizability. It was also performed to determine the consistency of the internal threshold employed by each reviewer in their evaluation of potential HFOs. In all cases, the HFO ratings made for each epoch by each reviewer were used as the G-study “measurement.”

#### Models

A nested mixed-effects model was used for the G-study analyses. The Image 1 in the Supplementary Material shows a Venn diagram depicting the primary effects and their interactions:

- Reviewer (*r*), the clinician evaluating the data for HFOs
- Dataset (*d*), the patient from whom the data were collected
- EventType (*t*), the type of event used to generate the epoch
- *e*:[*d* · *t*], the individual Epoch, nested within Dataset and EventType
- The interactions amongst the above effects

The generalizability theory model is expressed as:

$$\begin{array}{l} X_{rdte}=\mu +\nu_{r}+\nu_{d}+\nu_{t}+\nu_{r\cdot d}+\nu_{d\cdot t}+\nu_{r\cdot t}+\nu_{r\cdot d\cdot t}+\nu_{e:\left[d\cdot t\right]}+\nu_{r\cdot e:\left[d\cdot t\right]} \end{array}$$

where *X*_*rdte*_ is the HFO rating given by Reviewer *r* to Epoch *e* of Dataset *d* and EventType *t;* μ is the grand mean HFO rating; and ν_α_ is the score effect for any arbitrary effect α.

#### Objects of measurement

In generalizability theory, any one of the effects can be declared the “object of measurement,” while the other effects constitute the “facets” of variability. As the names imply, the object of measurement is what we are measuring, while the facets are effects to which we can attribute the variability of our measurements. The assignment of the object of measurement depends upon the question of interest. This study was designed to determine how well one could answer two questions:

1. What is the likelihood that Epoch *e*_*i*_ is an HFO?

2. What is the stringency of Reviewer *r*_*i*_ in evaluating Epochs for HFOs?

In order to address these questions, two separate objects of measurement were used. For Question 1, the apparent object of measurement was Epoch; however, since Epoch is nested within Dataset and Type, the object of measurement was actually *e*:[*d*·*t*]. For Question 2, the object of measurement was simply Reviewer (*r*). It is important to note the primary objective of the study is not to provide answers to these questions with the present sample sizes, but rather to quantify and predict how well the answers obtained would generalize to the population of epochs, reviewers, or patients.

#### Generalizability coefficient calculation

A minimum norm quadratic unbiased estimator (61, 62) was used to calculate the variance components, which were in turn used to derive the generalizability coefficients. Details regarding the calculations are provided in the Generalizability Theory Details section. A threshold of 0.8 was used to indicate high generalizability or interrater reliability (44).

#### Epoch generalizability—interrater reliability

For Epoch generalizability, the coefficient is a measure of how well the consensus HFO rating given by a set of Reviewers would generalize to the universe of potential Reviewers. It is an indicator of interrater reliability: a coefficient of 1.0 indicates that the HFO rating would generalize completely, while a coefficient close to 0.0 would indicate that it would not generalize. Optimizing this coefficient would reflect increasing the overall interrater reliability, and allow for the generation of a set of “universal” HFO Epoch evaluations.

#### Reviewer generalizability—internal reviewer threshold

The generalizability coefficient of the Reviewer object is a measure of how well the relative observed stringency of Reviewers would generalize to the universe of potential Datasets and Epochs. A coefficient approaching 1.0 indicates complete generalizability of Reviewer stringency, which would permit the confident relative ranking of Reviewers in terms of their probability of positively identifying an HFO of a given threshold. A low coefficient, on the other hand, would indicate that such a ranking could not be reliably made. Such rankings could be useful for clinical purposes, as in ensuring that a selected set of Reviewers would not be skewed toward a high or low stringency. They would be even more valuable for training purposes: Reviewers with a lower stringency could be trained to increase their internal threshold, while those with a higher stringency could be trained to decrease it.

### Decision study projections

In order to determine how the generalizability of each object of measurement would be affected by changes in the other facets, a decision study (D-Study) projection was performed for each object of measurement. The generalizability of the Epoch rating was projected across 5–300 Datasets and 2–20 Reviewers, and the generalizability of the Reviewer rating was projected across 1–3,000 Epochs and 5–300 Datasets. This enables us to predict how many datasets, reviewers, or epochs would be needed for us to achieve strong generalizability; in other words, it is used to predict the number of reviewers that would be needed for studies interested in determining the presence of HFOs, or the number of epochs and datasets that would be needed for studies interested in determining the internal stringency of reviewers.

### Generalizability theory details

The following section contains the details regarding the definitions and calculations involved in the derivation of the generalizability coefficients.

#### Variance components

The variance components (σ_*a*_^2^ for facet α) were estimated within SPSS. Specifically, the VARCOMP procedure was implemented using a minimum norm quadratic unbiased estimator [MINQUE; (61, 62)], with an intercept term and uniform weight assignment. These variance components were independent of the object of measurement.

The normalized variance components (σ_*A*_^2^ for facet α) were then computed by normalizing the variances component by the sample sizes (*n*_*a*_) of all effects in the given facet, regardless of whether the effects are crossed or nested. Sample formulae are illustrated below:

$$\begin{array}{l} \sigma_{A}^{2}=\frac{\sigma_{a}^{2}}{n_{a}}\;\sigma_{A\cdot B}^{2}=\;\frac{\sigma_{a\cdot b}^{2}}{n_{a}\cdot n_{b}}\;\sigma_{A:B}^{2}=\;\frac{\sigma_{a:b}^{2}}{n_{a}\cdot n_{b}} \end{array}$$

The sample size for the object of measurement was set to 1, so this process was repeated for each object of measurement.

#### Object and residual variances

The object of measurement variance ($\sigma_{\tau }^{2}$) is the variance attributable to the object of measurement. The object variances of all objects were estimated by summing the variance component of the object of measurement with those of any interactions between the object of measurement and fixed facets only:

$$\begin{array}{l} \sigma_{\tau }^{2}\left(e\right)=\sigma_{e:\left[D\cdot T\right]}^{2}\;\sigma_{\tau }^{2}\left(r\right)=\sigma_{r}^{2}+\sigma_{r\cdot T}^{2} \end{array}$$

The relative residual variance ($\sigma_{\delta }^{2}$) is the variance attributable to the interaction between the object of measurement and the random effects. The relative residual variances of all objects were estimated by summing the variance components of interactions between the object of measurement and at least one random facet:

$$\begin{array}{lll} \sigma_{\delta }^{2}\left(e\right) & = & \sigma_{R\cdot e\left[D\cdot T\right]}^{2}\\ \sigma_{\delta }^{2}\left(r\right) & = & \;\sigma_{r\cdot D}^{2}+\sigma_{r\cdot D\cdot T}^{2}+\sigma_{r\cdot E\left[D\cdot T\right]}^{2} \end{array}$$

The absolute residual variance ($\sigma_{\Delta }^{2}$) is the variance not solely attributable to the object of measurement. The absolute residual variances of all objects were estimated by summing the variance components of all facets excluded from $\sigma_{\tau }^{2}$:

$$\begin{array}{lll} \sigma_{\Delta }^{2}\left(e\right) & = & \sigma_{R}^{2}+\sigma_{D}^{2}+\sigma_{R\cdot D}^{2}+\sigma_{R\cdot T}^{2}+\sigma_{D\cdot T}^{2}+\sigma_{R\cdot D\cdot T}^{2}\\ +\sigma_{R\cdot e\left[D\cdot T\right]}^{2}\\ \sigma_{\Delta }^{2}\left(r\right) & = & \sigma_{D}^{2}+\sigma_{r\cdot D}^{2}+\sigma_{r\cdot T}^{2}+\sigma_{D\cdot T}^{2}+\sigma_{r\cdot D\cdot T}^{2}+\sigma_{E:\left[D\cdot T\right]}^{2}\\ +\sigma_{r\cdot E\left[D\cdot T\right]}^{2} \end{array}$$

#### Generalizability coefficients

Two generalizability coefficients may be estimated for each of object of measurement: A relative generalizability coefficient (ρ^2^) which describes how well the relative measurements amongst the objects generalize to the universe of facets, and an absolute dependability coefficient (φ^2^) which describes how well the absolute scores of the objects generalize to the universe. These may be estimated from the object variance as well as the relative or absolute residual variance, respectively, by:

$$\begin{array}{l} \rho^{2}=\frac{\sigma_{\tau }^{2}}{\sigma_{\tau }^{2}+\sigma_{\Delta }^{2}}\;\varphi^{2}=\frac{\sigma_{\tau }^{2}}{\sigma_{\tau }^{2}+\sigma_{\Delta }^{2}} \end{array}$$

In the present study, the relative measurements are of more interest compared to the absolute measurements: the likelihood of an epoch containing an HFO compared to other epochs, or the likelihood of a reviewer marking an HFO compared to other reviewers, are of greater interest than those likelihoods relative to some arbitrary value. As such, the following discussions are limited to the relative generalizability coefficients.

## Results

In total, 41,065 individual Epoch evaluations were made. A summary of the completed and missing evaluations is provided in the Supplementary Material (Data Sheet 1).

### Evaluation time

The trimmed mean evaluation time (excluding upper and lower 5% of values as outliers) for the six Reviewers were 7.15, 14.51, 4.32, 11.45, 4.93, and 8.43 s per Epoch, (overall mean = 7.43 s).

### Generalizability

The derivations of the G-study coefficients are outlined in Table 1. The generalizability coefficients were calculated to be 0.588 for Epoch and 0.983 for Reviewer.

Table 1Derivation of the generalizability coefficients for model considering all EventTypes.

**Table:** 

**(A)**							
		**Object: Epoch**			**Object: Reviewer**		
**Facet** α	$\sigma_{a}^{2}$	**n**_*a*_	$\sigma_{A}^{2}$	**Term**	***n**_*a*_*	$\sigma_{A}^{2}$	**Term**
*r*	1.134	6	0.189	Δ		1.134	τ
*d*	0.195	41	0.005	Δ	41	0.005	Δ
*r* · *d*	0.743	246	0.003	Δ	41	0.018	Δ, δ
*r* · *t*	0.638	18	0.035	Δ	3	0.213	τ
*d* · *t*	0.161	123	0.001	Δ	123	0.001	Δ, δ
*r* · *d* · *t*	0.553	738	0.001	Δ	123	0.004	Δ, δ
*e*:[*d* · *t*]	1.409		1.409	τ	7,872	0.000	Δ
*r · e*:[*d · t*]	5.925	6	0.988	Δ, δ	7,872	0.001	Δ, δ

**Table:** 

**(B)**							
**Object**	$\sigma_{\tau }^{2}$	$\sigma_{\delta }^{2}$	$\sigma_{\Delta }^{2}$	ρ^2^	φ^2^
Epoch	1.409	0.988	1.222	**0.588**	0.536
Reviewer	1.347	0.024	0.030	**0.983**	0.978

***(A)** Overview of variance components. Each row corresponds to the effect or interaction listed in the first column. The sets of columns outline the variance components (leftmost column set), as well as the normalized variance components where the object of measurement is set to Epoch (second column set) or Reviewer (third column set). For each object of measurement, the effective sample sizes (n_a_), normalized variance components ($\sigma_{A}^{\text{2}}$), and term(s) corresponding to the variance components are listed. **(B)** The object variances ($\sigma_{\tau }^{\text{2}}$), relative residual variances ($\sigma_{\delta }^{\text{2}}$), absolute residual variances ($\sigma_{\Delta }^{\text{2}}$), relative generalizability coefficients (ρ^2^), and absolute dependability coefficients (φ^2^), for each object of measurement. The relative generalizability coefficients are indicated in bold*.

### Decision study

#### Epoch generalizability

**Figure 2:**
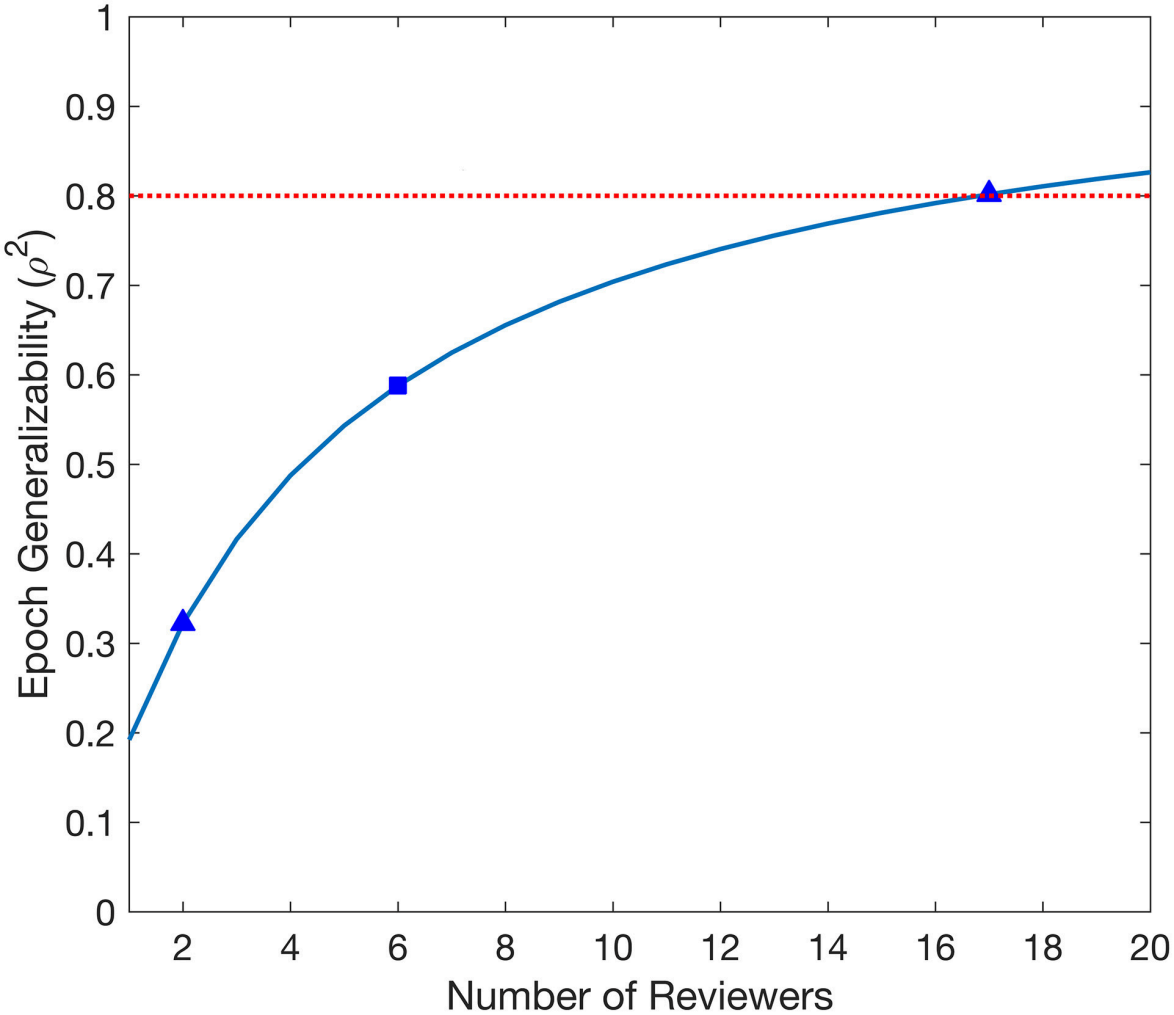
Decision study projections of the generalizability of the evaluated Epochs. The blue curve denotes the Epoch generalizability projections as a function of the number of Reviewers. The target threshold of 0.8 is indicated by the dashed red line. The blue square marks the Epoch generalizability achieved with the sample size used (6 Reviewers), while the blue triangles indicate Epoch generalizability projections referenced in the text.

The D-study projections for Epoch generalizability are displayed in Figure 2. The projections increased with the number of Reviewers, reaching the threshold of 0.8 with 17 Reviewers, and were independent of the number of Datasets.

#### Reviewer generalizability

**Figure 3:**
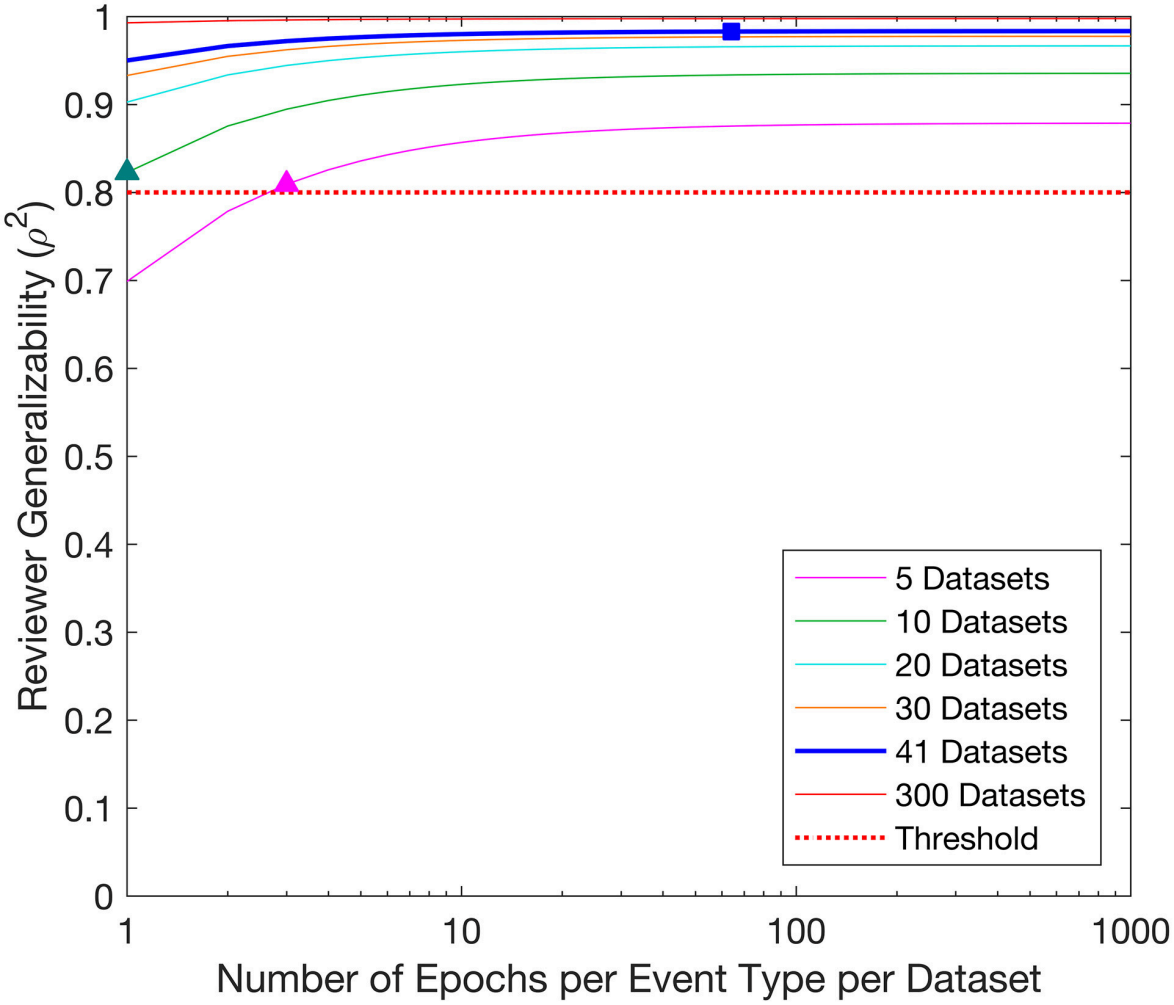
Decision study projections of the generalizability of the Reviewers. Each curve denotes the Reviewer generalizability projections for a given number of Datasets, as a function of the number of Epochs per EventType per Dataset. The target threshold of 0.8 is indicated by the dashed red line. The blue square indicates the Reviewer generalizability achieved with the sample sizes used in the study (64 Epochs per EventType, 3 EventTypes, 41 Datasets), while the colored triangles indicate Reviewer generalizability projections referenced in the text.

The D-study projections for Reviewer generalizability are displayed in Figure 3. The Reviewer generalizability projections increased with the number of Epochs and Datasets. A trend of diminishing returns was demonstrated, wherein the generalizability reached a plateau beyond which increases in the number of Epochs did not yield an appreciable increase in generalizability. For example, the threshold of 0.8 was achieved with 1 Epoch per EventType per Dataset in 10 Datasets, or with 3 Epochs per EventType per Dataset in 5 Datasets.

## Discussion

The generalizability studies reaffirm the poor interrater reliability typically observed between two reviewers (44), and the projections indicate that a prohibitive number of reviewers are required to achieve strong generalizability. The results also illustrate the strong Reviewer generalizability and the relatively small evaluation times within the novel epoched framework. Together, these findings provide further evidence for the need for a gold standard for HFO identification, and a framework for its establishment.

### Evaluation time

There was a notable variability amongst the Reviewers' mean evaluation times. This is unsurprising, as evaluation times also vary in other medical imaging applications (63, 64). Further investigation into the nature of the differences between Reviewers could assist the optimization of HFO evaluations for any given Reviewer.

It is equally unsurprising but notable that evaluation of HFOs using this supervised framework is substantially faster than continuous review. One of the Reviewers reported that prior to this study, he often required 8 h to review 5 min of continuous EEG data for HFOs in multiple channels. Using the trimmed mean evaluation time of 7.43 s, he would be able to evaluate 3876 Epochs in those same 8 h. Even considering our most active HFO patient, who exhibited 278.7 algorithmically-detected events per minute (across all event types, and all electrodes), this Reviewer would only have to evaluate 1394 Epochs from 5 min of data, which would take only 2.87 h. In the case of our second most active patient, he would only have to evaluate 350 Epochs (<45 min). Thus, he would complete the evaluations in substantially less time compared to continuous review in most cases. Furthermore, it is likely that he would have to evaluate only a fraction of the Epochs, further reducing the time burden.

This anecdotal evidence is further supported by a recent study reporting that approximately 5 h were required review 10 min of continuous EEG for spikes and ripples in a single channel derivation (27). This 2.5 h for 5 min of continuous data is somewhat less than the worst-case 2.87 h for epoched review noted above. However, the epoched review would provide markings for every channel, while the continuous review completed in that time constitutes markings for only a single channel.

### Epoch generalizability—interrater reliability

The D-study projections for Epochs predicted how many Reviewers would be required to achieve a desired Epoch generalizability. Each Epoch is selected from exactly one Dataset, and is entirely independent of other Datasets; therefore, D-study projections are only affected by the number of Reviewers. Only a moderate Epoch generalizability (0.588) was achieved with the six Reviewers used in this study. Notably, 17 Reviewers would be required to achieve a threshold of 0.8. This is a prohibitive amount for any individual epilepsy center, and would therefore not be feasible for the routine evaluation of any individual patient.

One solution to the requirement of such a large number of Reviewers would be to initiate a multi-center study, including at least 17 Reviewers, all evaluating the same Epochs. This would result in highly generalizable evaluations of individual Epochs, featuring both positive and negative HFOs of varying characteristics, with and without artifacts, constituting a benchmark set of universal HFO evaluations. This benchmark could be used as a reliable standard for testing any number of HFO detection or classification algorithms, or for the testing or training of Reviewers. The development of such a benchmark dataset is currently underway, featuring epileptologists from multiple centers across western Canada.

The Epoch generalizability for two Reviewers was estimated to be 0.322. Notably, this is in agreement with our previous study, which found poor interrater reliability when using a smaller dataset (44). This supports the use of the Epoch generalizability coefficient as a measure for interrater reliability, and provides further evidence against the use of agreement between two visual reviewers as a “gold standard” for HFO markings.

### Reviewer generalizability—internal reviewer threshold

The D-study projections for Reviewer predicted how many Epochs and Datasets would be required to achieve a desired Reviewer generalizability. For example, to achieve a Reviewer generalizability of 0.8, each Reviewer could evaluate 3 Epochs per EventType in 5 Datasets (3 × 3 × 5 = 45 evaluations), or 1 Epochs per EventType in 10 Datasets (1 × 3 × 10 = 30 evaluations). A study interested only in establishing a ranking of Reviewers by achieving a Reviewer generalizability of 0.8 could therefore be constructed with as few as 30 evaluations per Reviewer. Such a study could be completed for any given set of Reviewers, and would require an average time commitment of just under 4 min, assuming the trimmed mean observed Epoch evaluation time of 7.43 s.

Given this high Reviewer generalizability, there need not be 17 Reviewers to produce highly generalizable Epoch evaluations. Rather, the Epoch generalizability could be increased by increasing training with respect to the HFO evaluations. In the present study, training was limited to the presentation of an instructional video and companion document, which defined HFOs and provided clear examples. This training was designed to avoid influencing the internal threshold of the Reviewers. Conversely, training Reviewers to adjust their internal thresholds would decrease the variability between Reviewers (65–67), increasing the generalizability of their Epoch evaluations. Our group is currently conducting one such study using the principles of the Delphi method (68), whereby Reviewers first conduct the study with no *a priori* information, and then again using feedback information regarding the stringency of their evaluations relative to that of their peers. An increase in the Epoch generalizability could be expected with the Delphi method, which would decrease the number of Reviewers needed to obtain universal HFO evaluations for any given Epoch.

Such training procedures might facilitate the feasibility of on-site evaluation of patient data for HFOs as an informative component of the epilepsy pre-surgical workups. The proposed threshold training could also reduce the number of Reviewers needed to construct a benchmark dataset, or to further increase its generalizability. These possibilities should be thoroughly evaluated following the implementation of this Delphi study.

### HFO evaluation framework

Overall, this study has established a novel framework for efficient and controlled evaluation of HFOs. The Epoched design allows for the pre-selection of events based on any number of algorithmic criteria, and can be tailored to the particular application. It also ensures that all Reviewers can evaluate the exact same events of interest in the exact same order, in a manner that requires only an average of 10 s per evaluation. The evaluation software program provides Reviewers with an intuitive and versatile interface to complete any number of evaluations. It enables Reviewers to not only mark the presence of HFOs, but also the absence of HFOs, and the self-reported certainty in these determinations.

The present study has established that evaluations conducted using this supervised method are not sufficient to be used immediately in clinical practice. Rather, the framework itself may be extended to any number of automated detectors, to any number of reviewers, or to any form of standardized training, producing a comprehensive set of evaluations accompanied by descriptive information regarding the generalizability of the findings.

### External applicability

Notably, detection of electrographic signals such as HFOs are often performed using a visual review of a continuous record, or using an automated detector alone, rather than a supervised framework. However, the calculated generalizability coefficients and the predicted number of reviewers apply only to studies undertaken in this framework. It is entirely possible that HFO markings made on a continuous record would require fewer reviewers to achieve strongly generalizable findings, due to influences such as the additional context provided by the continuity of the record. Likewise, it is intuitive that the context provided by a continuous record may bias the markings—such as cases where clear or frequent HFOs in one channel preclude more ambiguous or occasional HFOs in another channel from being noticed. Furthermore, the sheer volume of continuous data may result in different reviewers focusing their search of HFOs on different channels or time windows, increasing the number of reviewers required to achieve strongly generalizable findings. Regardless of such points of speculation, it should be unambiguously emphasized that one cannot assume that HFO markings obtained through continuous review are generalizable—rather, it must be assumed that they are not generalizable, until it can be proven that they are.

The findings of this study are inherently applicable to the oscillations detected using two thresholds of an established detection algorithm, along with baseline EEG segments. The generalizability may differ as the threshold or method used to detect candidate HFOs varies, as might the clinical meaning of such oscillations. Further studies conducted within this framework may indeed be used to compare the meaning and generalizability of HFOs identified at various thresholds, in other frequency bands, or using different detection algorithms.

Alternatively, this framework could allow for the evaluation of HFO detection algorithms in several capacities, including comparing the performance of countless HFO detection algorithms against generalizable markings obtained by a large set of visual reviewers. A detection algorithm that is shown to be consistent with strongly generalizable visual markings could then be used as a standard, either alone or in conjunction with one or two reviewers, replacing the ongoing need for such a large number of visual reviewers.

Furthermore, while the waveforms of interest in this study were HFOs identified on intracranial EEG, studies have shown that other electrographic markers are subject to varying degrees of interrater reliability. It would be interesting to extend the methodology presented herein to other EEG markers independently of HFOs, such as epileptiform discharges, or even other electrographic markers, such as U waves on ECG, to determine the generalizability of those visual markings. It would also be interesting to apply this methodology to simultaneous EEG and magnetoencephalography, which has recent shown promising results for the source localization of HFOs and IEDs (69). The present study focused on the generalizability of HFO detection. As a next step, we are currently assessing how the algorithmically detected HFOs correlate with location of the seizure onset zone and post-surgical outcome.

### Limitations and future direction

The findings of this study establish a framework for further studies, which can efficiently reduce or eliminate many factors that currently limit conclusions that may be drawn in HFO studies.

One notable consideration is the degree of *a priori* training of the various Reviewers. All of the Reviewers in the present study were familiar with HFOs, and all were given both an instructional video and a short written document to help them become acquainted with the software used and the criteria used to evaluate HFOs in this study. However, the training was not exhaustive and was not designed to influence the internal threshold of the Reviewers with respect to HFO evaluations—only the clearest HFOs were presented as examples. It is reasonable to assume that two reviewers trained at the same center would be more similar than reviewers with different training backgrounds, and that therefore the markings made by two such reviewers would be more generalizable. However, our previous work found the pairwise interrater reliability between two similarly and extensively trained reviewers to be as poor as the overall interrater reliability (44). Furthermore, it is likely that across the global population of HFO reviewers, the individual training received would also vary greatly. Nonetheless, standardized interventional training such as the simple Delphi study described above may be effective in using feedback between evaluation sessions to encourage Reviewers to align their internal threshold to a desired target, thus increasing the generalizability of evaluations, whatever the background of any given Reviewer.

There are other confounding factors that may affect how representative our sample Epochs, Datasets, or Reviewers were, which could influence the generalizability. Additionally, Reviewers may be affected not only by *a priori* training, but also by fatigue; fortunately, the framework presented herein is designed to preclude fatigue, by reducing the time commitment of HFO evaluations, and future studies aimed at optimizing interrater reliability could further reduce the workload. Also, the Datasets or Epochs may be affected by time of day, admission date, and evaluation order: steps were made to control for time of day and admission date, by making the data selection across patients consistent, and the evaluation order was standardized for all Reviewers. Nonetheless the impact of any of these effects is unknown, but the proposed framework is well suited to study the effect sizes.

Another effect that may be addressed in future studies is that of reproducibility, which examines the variability in HFO evaluations of the same Epochs by the same Reviewers on multiple occasions. Such internal consistency is currently being assessed by incorporating multiple occasions into the aforementioned Delphi study of a large number of Reviewers.

## Conclusion

We have reaffirmed that the current practice in visual HFO identification, which uses the agreement between only two visual reviewers to identify HFOs, is unreliable due to significant variability between reviewers. We have also projected that the large number of visual reviewers required to produce reliable HFO markings would be an impractical barrier to undertaking visual HFO review in a clinical setting without first reducing the variability amongst reviewers.

We have also outlined a set of tools, including an evaluation program and a statistical framework, which may be used for training visual reviewers and tracking changes to interrater reliability, all while reducing the time burden of HFO analysis. Ongoing studies at our research center are using established training protocols within the framework presented herein to standardize the internal HFO threshold of visual reviewers, further reducing the time burden. It may alternatively serve as the basis of a multicenter study to construct a benchmark set of epochs that can serve as a new, highly generalizable gold standard, against which any number of HFO detection algorithms may be compared.

Ultimately, this study represents an important step toward the reconciliation of important but discordant findings from HFO studies undertaken with different sets of HFOs, and ultimately toward transitioning HFO analysis into a feasible and meaningful part of the epilepsy pre-surgical workup.

## Ethics statement

This study was carried out in accordance with the recommendations of the University of Calgary Conjoint Research Ethics Board with written informed consent from all subjects. All subjects were given written informed consent in accordance with the Declaration of Helskinki. The protocol was approved by the University of Calgary Conjoint Research Ethics Board.

## Author contributions

AS, DP, and PF contributed conception and design of the study. AS and PF performed the initial data collection. AS wrote the morphology detector. DP created the evaluation program. YA, JJ, NP, LB, CJ, and PF performed the primary data analysis. AS performed the statistical analyses. AS, DP, and PF interpreted the data. AS wrote the first draft of the manuscript. All authors contributed to manuscript review, and read and approved the submitted version.

### Conflict of interest statement

The authors declare that the research was conducted in the absence of any commercial or financial relationships that could be construed as a potential conflict of interest.
