# Supplementary material for: Generalizability of High Frequency Oscillation Evaluations in the Ripple Band
Source: Front Neurol. 2018 Jun 28;9:510. doi: 10.3389/fneur.2018.00510 (PMC6031752; doi:10.3389/fneur.2018.00510)
Supplement: Supplementary file 1 [file Data_Sheet_1.docx]

Supplementary Material

Generalizability of High Frequency Oscillation Evaluations in the Ripple Band

Aaron M. Spring, Daniel J. Pittman, Yahya Aghakhani, Jeffrey Jirsch, Neelan Pillay, Luis E. Bello-Espinosa, Colin Josephson, Paolo Federico*

*** Correspondence:** Paolo Federico: pfederic@ucalgary.ca

# Supplementary Figure

***Image 1***: Visualization of the model used for the generalizability theory studies. The model effects and their interactions are: Reviewer (*r*, red), Dataset (*d*, green), EventType (*t*, blue), *r · d* (yellow), *r · t* (magenta), *d · t* (cyan), *r · d · t* (white), *e*:[*d · t*] (dark grey), and *r · e*:[*d · t*] (light grey).

# Supplementary Data

## Missing Data Points

All six Reviewers were provided with the entirety of the data, which were to be evaluated in a pre-determined order. Given that 64 Epochs of each of three EventTypes were pseudorandomly selected from each of 41 patient Datasets, the total number of evaluations to be made by each Reviewer was 7872.

However, a variety of technical factors resulted in “missing data”, and the nature of the study precluded a recovery or a re-evaluation of these data points. As such, five of the six Reviewers evaluated fewer than 7872 total Epochs (7868, 7875, 7860, 7680, and 1920).

Four Reviewers experienced minor technical difficulties with the evaluation software, wherein some evaluations were not correctly written to disk. These evaluations could not be redone, as the order of the evaluations is a confounding factor in the study. Similarly, the context could not be recreated, as the effect of re-evaluating the same Epochs is unknown. The technical difficulties occurred for different Epochs for each Reviewer, resulting in missing data for at most one of the four Reviewers in any affected Epoch. It should be emphasized that the Reviewers did see these Epochs, and did evaluate them, so the following evaluations were not affected (whereas if the Reviewers had not seen the Epochs at all, the context for the subsequent evaluations may have been artificially affected).

The sixth Reviewer encountered no such errors during the evaluation of the first ten Datasets, but experienced substantial technical and logistical difficulties preventing the evaluation of the remaining 31 Datasets. Due to overlap between the missing Datasets from the sixth Reviewer and the Epoch errors from the other Reviewers, a total of 207 Epochs were only evaluated by four Reviewers, but no Epochs were evaluated by fewer than four Reviewers. Again, given that all of the Epochs that the sixth Reviewer was able to evaluate were presented in the correct order, and that all missing Datasets were to have been evaluated later, the context of the existing evaluations were not affected by the missing data.
