# Supplementary figures and images for: Generalizability of High Frequency Oscillation Evaluations in the Ripple Band
Source: Front Neurol. 2018 Jun 28;9:510. doi: 10.3389/fneur.2018.00510 (PMC6031752; doi:10.3389/fneur.2018.00510)

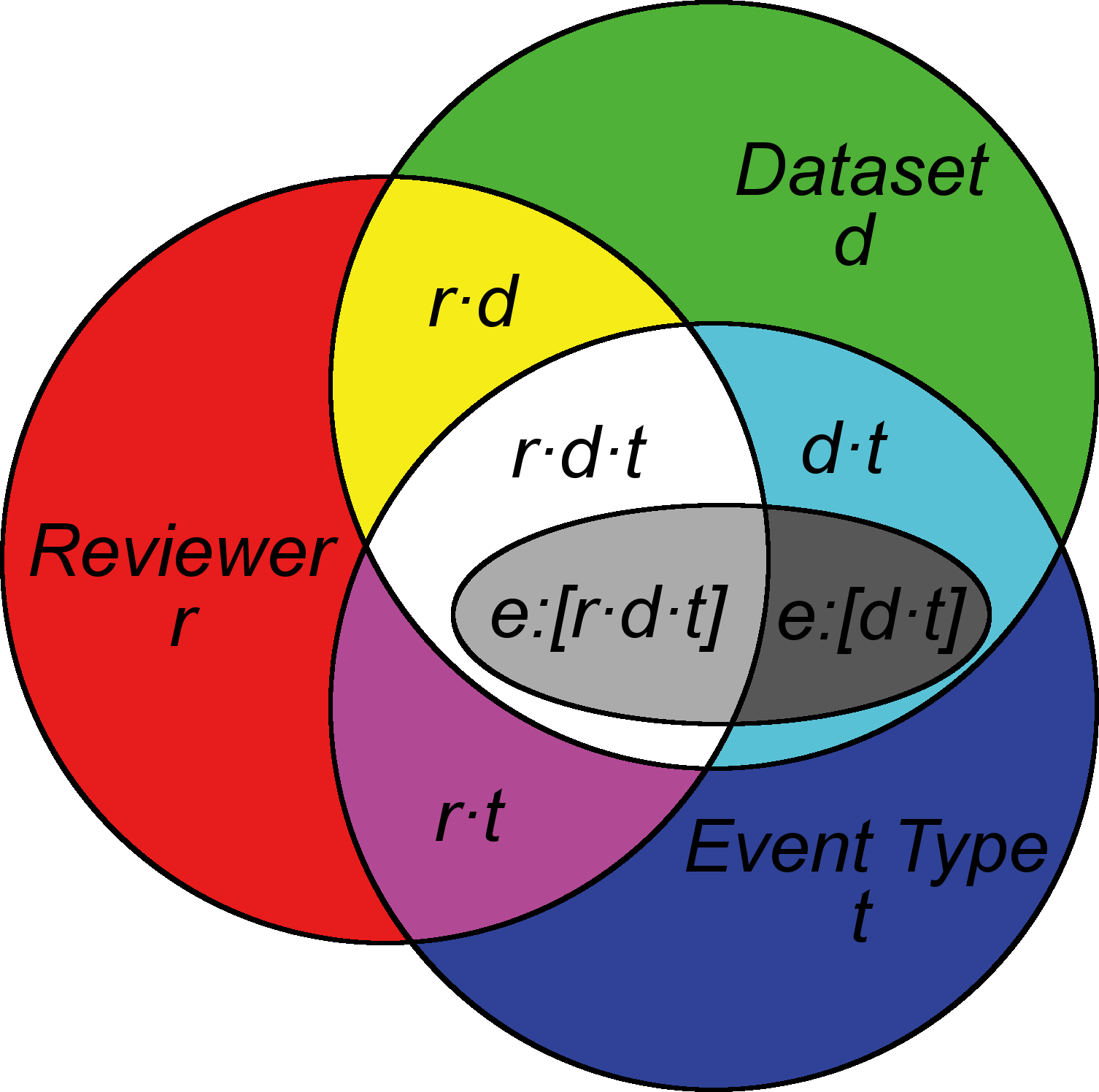

Supplement: Supplementary file 2 [file Image_1.TIFF]
